# Palmieri’s double suture skin repair: a new double suture approach to cases of skin cancer and ulcerative lesions

**DOI:** 10.1186/1477-7819-12-70

**Published:** 2014-03-28

**Authors:** Beniamino Palmieri, Simone Grappolini, Barbara Fiamengo, Tommaso Iannitti

**Affiliations:** 1 Department of General Surgery and Surgical Specialties, University of Modena and Reggio Emilia Medical School, Surgical Clinic, Modena, Italy; 2 Poliambulatorio del Secondo Parere, Modena, Italy; 3 Department of Plastic Surgery, Istituto Clinico Humanitas, Milan, Italy; 4 Department of Pathology, Istituto Clinico Humanitas, Milan, Italy; 5 School of Biomedical Sciences, University of Leeds, Mount Preston Street, Garstang building, Leeds LS2 9JT, UK

**Keywords:** Double suture, Ulcer, Cancer, Skin, Lesion

## Abstract

We describe two case reports to assess the efficacy of a new method suitable to close small-sized pressure ulcers and cancer-related skin lesions.

## Background

Breast implant reconstruction is a common and safe procedure after bilateral subcutaneous mastectomy or cosmetic treatments, but complications, like pressure ulcers or fistula formation, are reported in some cases [1,2]. Usually, at the beginning of the process, the fluid oozing from the skin wound is not contaminated, but, suddenly, bacterial or fungal colonisation begins [3,4], and removal of the prosthesis is mandatory in some cases [5]. Thus a very quick and careful attempt to primarily close the wound could avoid a further and expensive operation, but a specific suture strategy is required to reinforce the skin texture and allow the periprosthetic capsule and skin to close the gap definitely, avoiding the risk of transfixion injury to the prosthesis. Our technique has been conceived to fulfil these safety and effectiveness criteria, and it can also be extended to the repair of other circular skin defects in other anatomic districts such as head, neck, trunk arms or legs, whenever elasticity and pliability of the skin surface has a good compliance, especially in oncoplastic surgery. In this study, we propose a two-layer skin suture to double the thickness of the scar covering the original ulcer edges. This goal is achieved by a first very thin purse-string aesthetic suture which does not transfix the underlying prosthesis, followed by a linear row of several (6/0 braided and coated synthetic, mid-term absorption suture with precision point cutting edge 3/8 needle) thin contiguous stitches to reduce the amount of bulky collagen reaction, with final strong overlapping fusion of the skin margins.

## Case presentation

### First case presentation

**Figure 1:**
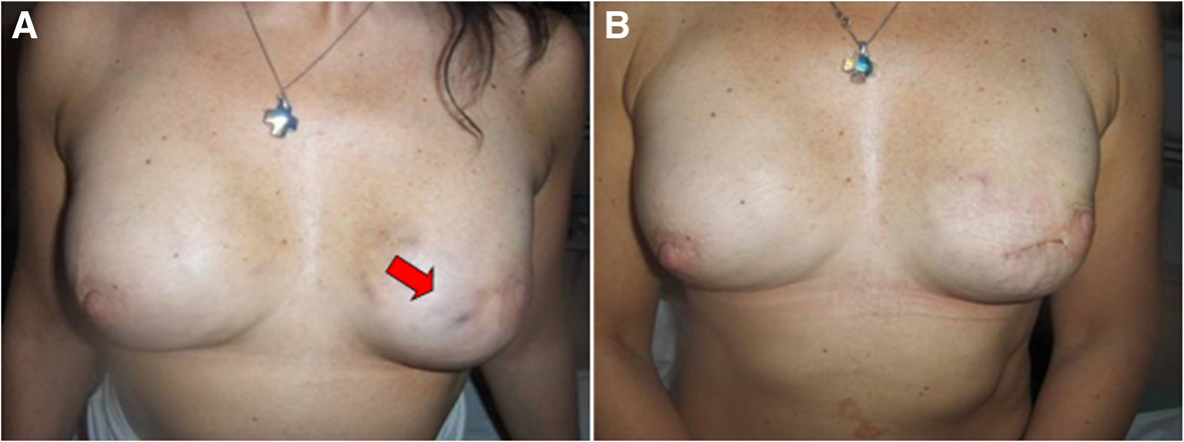
**First case report. A)** Small decubitus sterile serum oozing wound in the lower inner left quadrant of the breast (arrow) prior to surgical procedure; **B)** Appearance of the breast after surgical procedure: a very fine horizontal suture line hinders the previous 6/0 ophthalmic needle purse string around the pressure ulcer circumference.

A 42-year-old woman underwent delayed nipple-sparing mastectomy, tissue expanders and submuscular and partial subcutaneous prosthesis. She developed a small pressure ulcer (4 × 4 mm) caused by friction in the lower inner left quadrant of her left breast (Figure 1A) oozing some serous fluid which was cultured and did not display any bacterial growth. This pressure ulcer was due to the weight of the silicon prosthesis applied to the patient’s tense breast skin.

The patient came to the Poliambulatorio del Secondo Parere (Modena, Italy), signed the informed consent and was immediately scheduled for prosthesis change in 2 weeks’ time to undergo a primary closure of pressure ulcer with our ‘fine thread technique’ and evaluate the intraoperatively harvested newly-formed scar by histological analysis of hematoxylin and eosin (H&E) standard tissue stain.

### Suturing technique (Palmieri’s suture)

A 6/0 suture (GLICOFIL® LAC, Assut Europe, Italy) with a double precision cutting point needle was carefully used to perform a purse string around the wound just beneath the deep dermis, without injuring the prosthesis shell. The thread was tied, completely sealing the lumen of the pressure ulcer, thus preventing any leakage. Two horizontal lines, 3 cm long and 1 cm apart from the upper and lower pole of the sutured circumference, were then scraped with a blade No. 15 up to the deep dermis to expose the microvascular dermal network as newly-formed wound lips. A second layer of 5/0 nylon stitches, with single precision point cutting edge 3/8 circle needle (ASSUNYL®, Assut Europe, Italy), at 2.5 mm intervals, was delivered to join the two lips and bury the previously performed purse string (Figure 1B). An adhesive skin closure (3M™ Steri-Strip™; 3M Italia Spa, Milan, Italy) reinforced the suture line, and polyurethane adhesive films covered the operated area. The patient received 1 g ampicillin three times a day for 3 days. No further medication was given to the patient until the moment of the final surgery.

### Surgical procedure

The procedure was performed 12 days after the suturing of the pressure ulcer in order to verify the new suture healing outcome from the histological point of view and state the feasibility and effectiveness of our method (less invasive than removing and replacing the prosthesis and finally repairing the skin defects).

Under general anesthesia, the adhesive skin closure over the suture was removed and the sutured skin segment was resected with the knife on a 3 cm long and 1.2 cm wide segment which was processed for histology. Then the prosthesis was delivered through the previous incision, the cavity was irrigated with saline and povidone-iodine for three times and a new prosthesis was inserted. The deep subcutis was reinforced with strips of the prosthesis collagen capsule, and the skin wound was sutured intradermally.

### Histological findings

**Figure 2:**
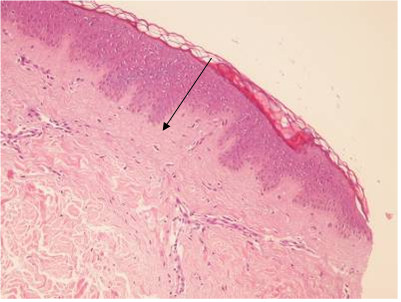
**Dermal fibrosis and perfect fusion of artificially created wound edges by means of small densely performed interrupted suture stitches (arrow).** (H&E; 100x).

**Figure 3:**
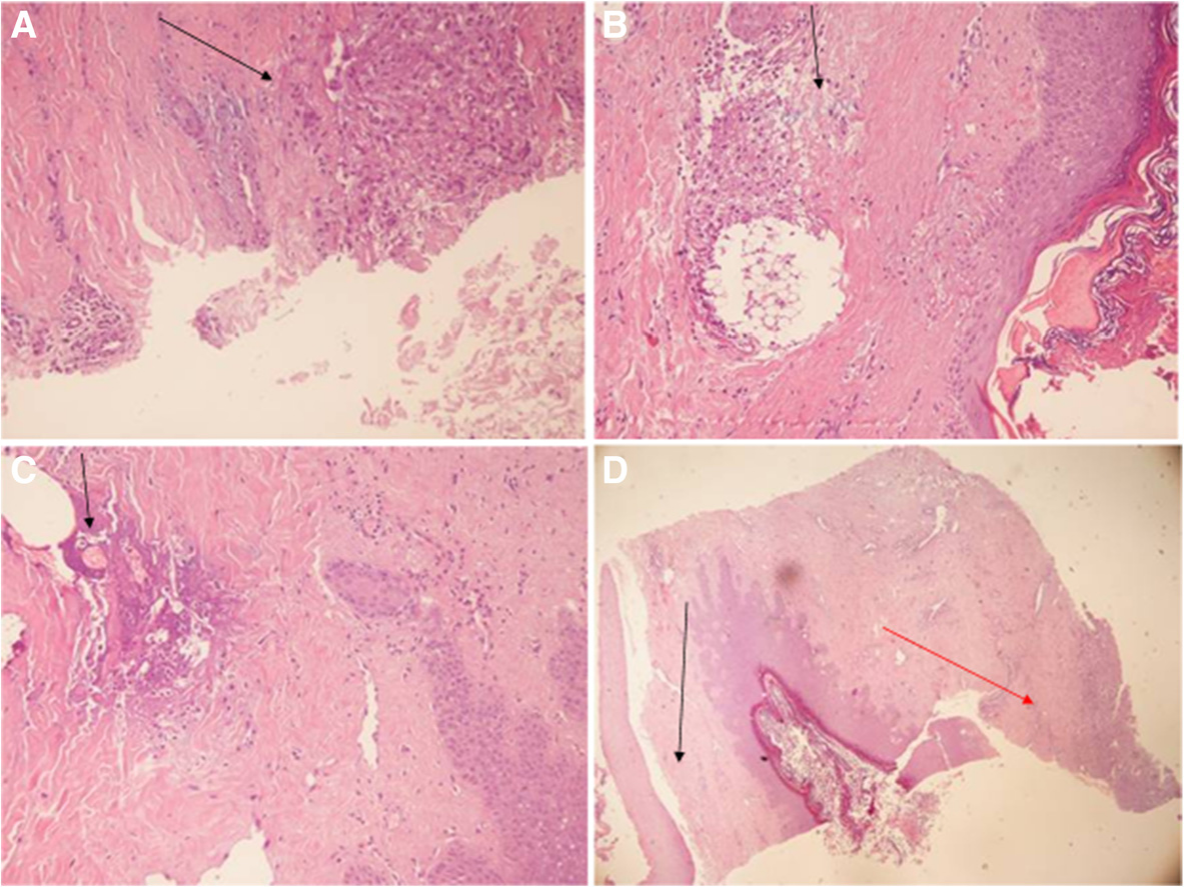
**Double suture histological evaluation. A)** Deep inflammatory reaction on the prosthesis borderline, contiguous to the sealed pressure ulcer, whose track is completely absent (arrow; H&E; 150x); **B)** Superficial foreign body reaction to the nylon stitches which have produced strong collagen reaction and doubled the skin thickness over the pressure ulcer (arrow; H&E; 100x); **C)** The absorbable purse-string suture buried around the pressure ulcer circumference with moderate foreign body inflammatory reaction (arrow; H&E; 100x); **D)** Overview of superficial plane (black arrow) and deep plane (red arrow) with a nylon intermingled suture and complete restoration of a thick dermal layer. The pressure ulcer track is not visible. (H&E; 100x).

The histological findings confirmed the complete functional success of the double suture. In fact, the buried wound was completely obliterated and replaced by young collagen spiraliform tissue with some chronic inflammatory cells in between. The purse-string stitch remnants were easily identified and fragmented (Figure 2). In the cut specimen, the second linear suture, sealing the two horizontal artificially-induced (blade scarification on both sides) wound lips, was identified as well, and showed a further strong, young collagen layer, with transversal well-arranged bundles increasing the overall dermis thickness. In this second plane, the chronic inflammatory cells were scantier. This second layer was quite firm and resistant and still suture debris could be identified among the collagen fibres (Figure 3A, B, C, D).

### Second case presentation

**Figure 4:**
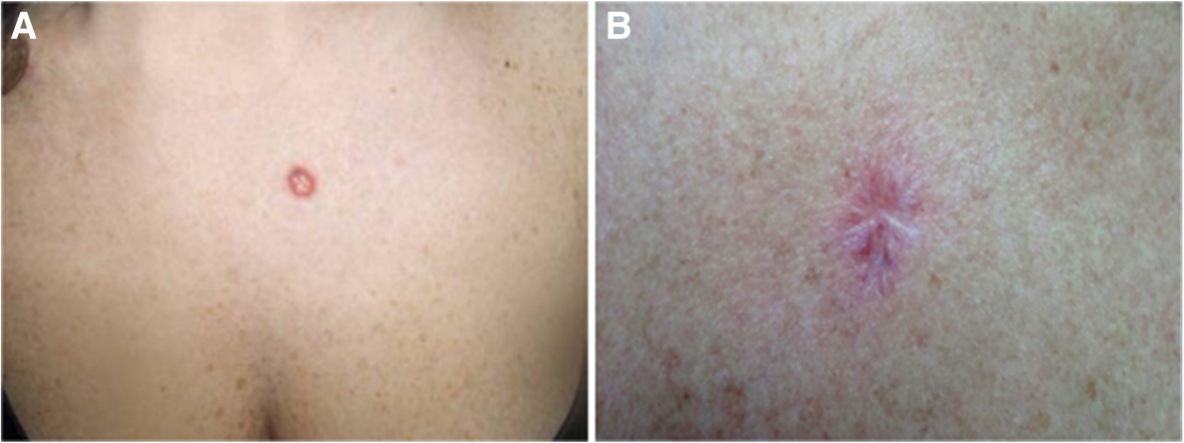
**Second case report. A)** Presternal basalioma of the skin; **B)** Final appearance of the scar after surgical procedure; the radial vectors of the purse-string suture are still visible; the upper layer of interrupted stitches gives protection to the deep scar.

Since 1940, skin tumours were often removed or debulked by excision or curettage. Nowadays, Mohs’ surgery for basal cell carcinoma (BCC) is widely recommended by dermatologists as first line surgical therapy for skin tumours [6]. Here we show that the Palmieri’s suture can also be applied to cases of lesions deriving from skin tumours. A 55-year-old woman came to the Policlinico del Secondo Parere (Modena, Italy) for the sudden appearance of a skin lesion in the presternal region**,** which was diagnosed as melanoma (Figure 4A). She had no family history of cancer, and was professionally exposed to the sun for 30 years.

We operated on her according to the following procedure: a 2/0 braided and coated synthetic absorbable purse-string suture (GLICOFIL® LAC, Assut Europe, Italy) was performed around the tumour area in the surrounding healthy skin. The second step was the excision of the skin lesion with a portion of the free surrounding margins. Immediately after, the purse-string suture was tensed and tied in order to reduce the scar area as much as possible. A second interrupted row of 3/0 Glicofil stitches (GLICOFIL® LAC, braided mid-term absorption, Assut Europe, Italy) covered the operated area according to our double suturing technique, as described above. The procedure was successfully performed with a reasonably good cosmetic outcome (Figure 4B) decided by the surgeon and in accordance with the patient. The melanoma was not present at the 48-month follow-up.

## Conclusion

There is a growing interest in developing new wound closure methods and materials [7]. The present case reports show the rationale and histological findings of a new small suture, named Palmieri’s suture, aiming to increase the thickness of the dermis to manage and close any uncomplicated skin lesions or pressure ulcers. We support the clinical use of this procedure especially for not infected or contaminated breast prosthesis, small-sized pressure ulcers as it can improve the skin resistance and bury the ‘locus minoris resistentiae’ of the ulcerated tegument under a thick collagen layer. In the dermatological/oncological surgery field, this technique is very useful because it reduces the size of the suture with a more appealing and less devastating cosmetic appearance. Our method differs from those previously described for two main reasons: (1) very thin aesthetic sutures are required either in the purse-string step to transfix an adequate portion of the dermis, without tearing the needle track or injuring the underlying prosthesis or in the superficial interrupted suture row where the densely applied nylon ophthalmic stitches produce a homogenous collagen layer with a strong but fine bundle network; (2) the second horizontal suture, covering the previous purse string, is performed by plication of the surrounding skin, following previous blade scraping of the microvascular dermal network of the skin margins. This procedure allows healing of the newly-formed opposite wound lips in a final resistant dermal scar. As a matter of fact, we did not find any buried remnants of the epidermal layers or adnexa at histological analysis where the dermal thickness was doubled by the skin suture overlying the ulcerated skin margins whose epidermal layer had previously been scraped out by the blade. This expedient differentiates Palmieri’s suture from those previously described, such as the schematically drawn suture by Fife and Alam [8]. Our technique fulfils the criteria of an easy and quick learning curve and strong effective skin consolidation, which are specifically required in prosthetic breast surgery. The proper size of the needle and the thread chemical composition make the difference and must be carefully evaluated to avoid prosthesis perforation, skin tearing and suture failure.

## Consent

Written informed consent was obtained from the patients for the publication of this article and any accompanying images.

## Competing interests

The authors declare that they have no competing interests.

## Authors’ contributions

BP, SG, BF and TI contributed equally to this work. All authors read and approved the final manuscript.

## Acknowledgement

None.
